# Increasing lipid production in *Chlamydomonas reinhardtii* through genetic introduction for the overexpression of glyceraldehyde-3-phosphate dehydrogenase

**DOI:** 10.3389/fbioe.2024.1396127

**Published:** 2024-04-19

**Authors:** Sung-Eun Shin, Hyun Gi Koh, Kyungmoon Park, See-Hyoung Park, Yong Keun Chang, Nam Kyu Kang

**Affiliations:** ^1^ Department of Chemical and Biomolecular Engineering, Korea Advanced Institute of Science and Technology (KAIST), Daejeon, Republic of Korea; ^2^ Department of Biological and Chemical Engineering, Hongik University, Sejong, Republic of Korea; ^3^ Department of Chemical Engineering, College of Engineering, Kyung Hee University, Yongin, Republic of Korea

**Keywords:** microalgae, *Chlamydomonas reinhardtii*, lipid, biofuels, glyceraldehyde-3-phosphate dehydrogenase, metabolic engineering, transcriptomics

## Abstract

Microalgae, valued for their sustainability and CO_2_ fixation capabilities, are emerging as promising sources of biofuels and high-value compounds. This study aimed to boost lipid production in *C. reinhardtii* by overexpressing chloroplast glyceraldehyde-3-phosphate dehydrogenase (GAPDH), a key enzyme in the Calvin cycle and glycolysis, under the control of a nitrogen-inducible NIT1 promoter, to positively impact overall carbon metabolism. The standout transformant, PNG#7, exhibited significantly increased lipid production under nitrogen starvation, with biomass rising by 44% and 76% on days 4 and 16, respectively. Fatty acid methyl ester (FAME) content in PNG#7 surged by 2.4-fold and 2.1-fold, notably surpassing the wild type (WT) in lipid productivity by 3.4 and 3.7 times on days 4 and 16, respectively. Transcriptome analysis revealed a tenfold increase in transgenic GAPDH expression and significant upregulation of genes involved in fatty acid and triacylglycerol synthesis, especially the gene encoding acyl-carrier protein gene (*ACP*, Cre13. g577100. t1.2). In contrast, genes related to cellulose synthesis were downregulated. Single Nucleotide Polymorphism (SNP)/Indel analysis indicated substantial DNA modifications, which likely contributed to the observed extensive transcriptomic and phenotypic changes. These findings suggest that overexpressing chloroplast GAPDH, coupled with genetic modifications, effectively enhances lipid synthesis in *C. reinhardtii*. This study not only underscores the potential of chloroplast GAPDH overexpression in microalgal lipid synthesis but also highlights the expansive potential of metabolic engineering in microalgae for biofuel production.

## 1 Introduction

Petroleum has long been regarded as an indispensable resource for producing fuels and chemicals. However, the persistent reliance on fossil fuels has led to significant environmental issues. These challenges underscore the urgent need for renewable and sustainable energy alternatives (Narayanan, 2024). Microalgae have gained attention as potential biofuel feedstocks because they have high photosynthetic productivity and CO_2_ conversion rates, require less land, may not compete with food sources, and can generate various value-added products (Kumar et al., 2023; Sun et al., 2023; Liu et al., 2024; Praveena et al., 2024).

*Chlamydomonas reinhardtii*, a unicellular biflagellate green alga, has been a focal point of research for several decades. Its high growth rate, capacity for sexual reproduction, and ease of cultivation have enabled *C. reinhardtii* to emerge as a microalgal model strain in both basic physiological research and applied biotechnology (Masi et al., 2023; Pessoa et al., 2023). Indeed, research on *C. reinhardtii* has significantly advanced the understanding of algal metabolism and has developed molecular tools and techniques for genetic engineering (Milito et al., 2023; Perozeni and Baier, 2023).

Recent research has focused on producing lipids from microalgae, which are vital for biodiesel production (Liu et al., 2024). This interest intensified following the discovery of starchless mutants in *C. reinhardtii*, which, unlike their wild type (WT) counterparts, accumulate significant amounts of lipids, primarily triacylglycerols (TAGs), instead of starch. In addition, it has been found that microalgae produce lots of lipids under various stress conditions, such as nutrient depletion or osmotic stress (Maltsev et al., 2023; Polat and Altinbas, 2023; Singh et al., 2023). Based on these results, there has been a growing interest in metabolic and cultivation engineering to enhance carbon flux towards lipids from CO_2_ (Dasan et al., 2023).

Typically, the overexpression of genes involved in fatty acid synthesis has been mainly conducted, such as acetyl-CoA carboxylase (ACCase), 3-ketoacyl-ACP synthase (KAS), fatty acid synthase (FAS), and pyruvate dehydrogenase kinase (PDK) (Kang et al., 2022). Metabolic engineering of the TAG synthesis pathway has also been explored by regulating the key genes, including glycerol-3-phosphate dehydrogenase (GPDH), glycerol-3-phosphate acyltransferases (GPAT), lysophosphatidic acid acyltransferases (LPAAT), phosphatidate phosphatase (PAP), and diacylglycerol transferase (DGAT) (Kang et al., 2022; Koh et al., 2023). Enhancing the Calvin cycle, gluconeogenesis, and glycolysis have also been studied to increase carbon flux towards lipids (Koh et al., 2019). In photosynthetic organisms such as higher plants and algae, those processes are mainly conducted in chloroplasts (Plaxton, 1996; Johnson and Alric, 2013).

Although not directly involved in lipid synthesis, glyceraldehyde-3-phosphate dehydrogenase (GAPDH) plays a crucial role in fatty acid synthesis in oleaginous species by supplying NADPH, which is essential for lipid synthesis. Theoretically, GAPDH can provide 1.16 moles of NADPH out of the 16 moles needed to synthesize 1 mole of C18-fatty acyl-CoA (Ratledge, 2014). Wang et al. demonstrated that overexpressing endogenous GAPDH in the oleaginous filamentous fungus Mortierella alpina resulted in a lipid content increase of 13–23%, while downregulation of these enzymes led to a significant reduction in biomass (Wang et al., 2020). Moreover, GAPDH is known to participate in various processes including transcriptional activation, cellular apoptosis, signal transduction, and abiotic stress resistance (Nicholls et al., 2012; Zhang et al., 2017). For instance, the fungus *Aspergillus nidulans* accumulated more glycerol by *GAPDH* gene expression under osmotic stress (Redkar et al., 1998). Additionally, overexpressing GAPDH from oyster mushrooms (*Pleurotus sajor-caju*) in potato plants improved salt tolerance, leading to enhanced productivity (Jeong et al., 2001).

However, the function of GAPDH may differ based on its localization. Cytoplasmic GAPDH is hardly involved in the Calvin cycle or chloroplastic glycolysis, which occurs within the chloroplast. This study investigates a chloroplast-targeted GAPDH that plays a dual role in both the Calvin cycle and chloroplastic glycolysis. Chloroplast GAPDH facilitates the reversible conversion of glyceraldehyde-3-phosphate (GAP) to 1,3-biphosphoglycerate (1,3-BPG). During CO_2_ fixation in the Calvin cycle, GAPDH converts 1,3-BPG to GAP, allowing carbon from fixed CO_2_ to undergo gluconeogenesis and glycolysis within the chloroplast. Conversely, in chloroplastic glycolysis, GAPDH reverses this process. Thus, chloroplast GAPDH potentially impacts both photosynthesis and glycolysis, influencing lipid and biomass production fluctuations. Consequently, we hypothesize that overexpressing chloroplast GAPDH in *C. reinhardtii* could significantly enhance CO_2_ fixation and lipid accumulation, especially under conditions of artificial stress such as nutrient deprivation.

The objective of this study was to enhance lipid productivity by overexpressing the chloroplast *GAPDH* gene under the control of an inducible nitrate reductase (NIT1) promoter in *C. reinhardtii*. The mutant strain was cultivated under nitrogen starvation to enhance GAPDH expression and stimulate lipid production. To understand the mechanisms behind this lipid enhancement, genome and transcriptome analyses of the transformant was conducted. The findings suggest that overexpression of chloroplast GAPDH positively affects lipid production under nitrogen-deprived conditions. These results indicate that GAPDH overexpression could be a viable approach for enhancing lipid production in microalgae.

## 2 Materials and methods

### 2.1 Microalgal strains and culture conditions

*Chlamydomonas reinhardtii* CC-124, obtained from the Chlamydomonas Resource Center at the University of Minnesota (http://www.chlamycollection.org/), was used as the wild type (WT). Strains were maintained on Tris-acetate-phosphate (TAP) agar plates, which consisted of 2.42 g/L Tris, 0.375 g/L NH_4_Cl, 0.1 g/L MgSO_4_·7H_2_O, 0.05 g/L CaCl_2_·2H_2_O, 0.0108 g/L K_2_HPO_4_, 0.0054 g/L KH_2_PO_4_, 1 mL/L pure glacial acetic acid (>99%), and 1 mL/L Hutner’s trace elements (50 g/L Na_2_EDTA·2H_2_O, 22 g/L ZnSO_4_·7H_2_O, 11.4 g/L H_3_BO_3_, 5.06 g/L MnCl_2_·4H_2_O, 1.61 g/L CoCl_2_·6H_2_O, 1.57 g/L CuSO4·5H_2_O, 1.10 g/L (NH_4_)_6_Mo_7_O_24_·7H_2_O and 4.99 g/L FeSO_4_·7H_2_O) at 25°C under continuous illumination of 50 μmol m^-2^ s^-1^. Cells were cultivated in 250 mL of TAP medium in 500-mL Erlenmeyer flasks plugged by sili-stopper (Shinetsu, Japan) at 25°C with shaking (120 rpm) under continuous fluorescent light (120 μmol m^-2^ s^-1^). For nitrogen starvation, cells were cultured with fresh nitrogen-depleted TAP medium. The cell growth was determined by OD_750 nm_, cell density (in cells/mL), and dry cell weight (DCW). OD_750 nm_ was measured by UV/Vis spectrometer (Shimadzu Co., Japan). DCW was estimated by filtering an aliquot of cultured cells with GF/C filter paper (Whatman, United States of America), drying at 105°C overnight, and weighing on a fine scale. Cell density (immobilized by 0.25% (w/v) iodine in ethanol) was counted with Cellometer AutoT4 (Nexcelom Bioscience, United States of America). Chemicals were purchased from Merck-Sigma-Aldrich unless otherwise specified.

### 2.2 Vector construction

**FIGURE 1:**
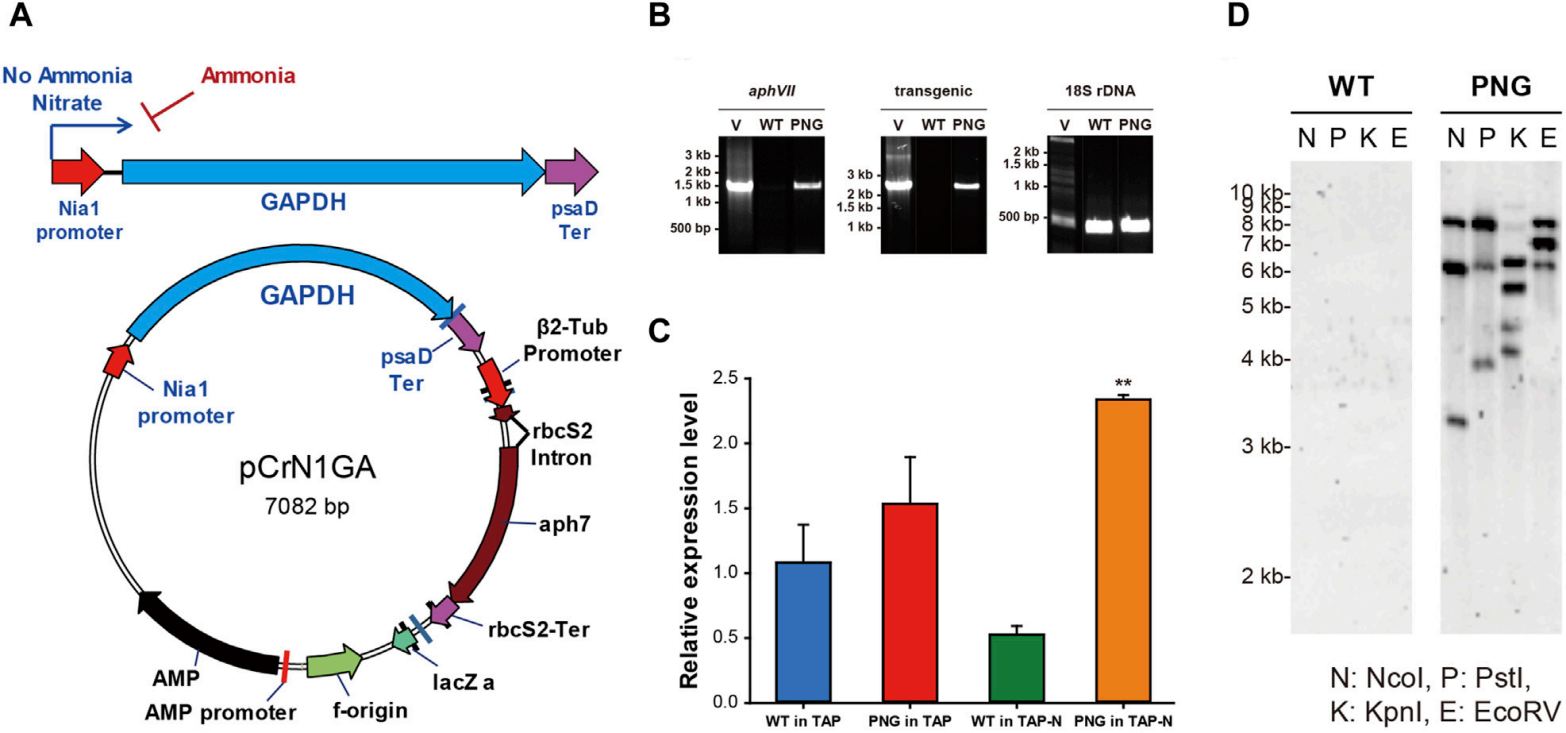
Vector construction of pCrN1GA and validation of successful gene integration and expression in PNG transformant **(A)** Schematic map of the vector pCrN1GA. **(B)** PCR confirmation of the integrated pCrN1GA vector sequences into WT and PNG transformants. The PCR products for the hygromycin resistance region (*aphVII*), the transgenic region extending from the NIA1 promoter to the chloroplastic *GAPDH* gene, and the 18S rDNA were 1,375 bp, 2,228 bp, and 380 bp, respectively. Specific details of the primers used are listed in Supplementary Table S1. **(C)** Expression of chloroplastic *GAPDH* in PNG transformants under nitrogen starvation. qRT-PCR analysis was performed on chloroplastic GAPDH using SHK GAPDH_F and SHK GAPDH_R primers at 6 h post-induction. Error bars represent standard errors from three independent experiments. Significant differences, as determined by the Student's *t*-test, are indicated by asterisks (*p < 0.05, **p < 0.01, ***p < 0.001). **(D)** Southern blotting of PNG transformant. The genomic DNAs were digested with several restriction enzymes and then hybridized with 387 bp probes of hygromycin-resistant genes. N: *Nco*I, P: *Pst*I, **(K)** *Kpn*I, and **(E)** *EcoR*V.

In order to overexpress chloroplast *glyceroaldehyde-3-phosphate dehydrogenase* (gapA) (*GAPDH*; GenBank accession: L27668), the inducible promoter of *nitrate reductase* (*NIT1*; GenBank accession: AF203033) (Loppes and Radoux, 2002) was used. Both the NIT1 promoter and the GAPDH gene were amplified from the genomic DNA of *C. reinhardtii* using the primers NIT_F/NIT_R for the NIT1 promoter and primers GF/GR for the GAPDH gene, respectively (Supplementary Table S1). The GAPDH gene contains a 22 bp long signal peptide and 5 introns in its sequence, which were not modified during the cloning procedure. The obtained PCR amplicons were cloned into the vector pCr102 carrying a hygromycin-selectable marker (*aphVII*) (Park et al., 2012). The psaD promoter in the pCr102 was replaced by the NIT1 promoter by digestion with *Kpn*I/*Hind*III, and the amplified *GAPDH* gene was inserted into the multi-cloning sites (MCS) by digestion with *Spe*I/*Sma*I. The vector was constructed by a DNA ligation kit (TaKaRa, Japan) and named pCrN1GA (Figure 1A).

### 2.3 Glass bead transformation and transformants selection by colony PCR

The recombinant vector was transformed to *C. reinhardtii* by glass bead methods, according to previously published methods (Kindle, 1990). The pCrN1GA vector was linearized by *Xba*I, purified, and concentrated to 1 μg/μL. Cells grown to the stationary phase were harvested and treated with autolysin under dark conditions for 3 h with agitation (60 rpm). Protoplast cells were mixed with 1 μg of vector, 100 μL of 20% polyethylene glycol, and 300 mg of sterile glass beads (Sigma, United States of America) and vortexed for 30 s on a vortex mixer (Fisher Scientific, United States of America). Transformed cells were recovered for 16 h in a TAP medium with agitation (120 rpm) under continuous low light. Transformant cells were selected on TAP agar plates containing 15 mg/L of hygromycin B (Thermo Fisher Scientific, United States of America). After 1 week, hygromycin-resistant colonies were isolated, and we conducted PCR verification of the following: the presence of the *aphVII* gene (using primers aph72_L and aph72_R); the presence of the transgenic *NIT1* and *GAPDH* gene (using primers NIT_F and GR; primers NIT_F and GMR); and proper amplification of the 18S rDNA (using primers SR6_F and SR9_R) as a positive control (Supplementary Table S1). Crude DNA of WT and transformants was extracted by Instagene Matrix (Bio-Rad, United States of America) following the manufacturer’s instructions, and used as a template for genomic PCR. 2× EF-taq PCR Pre-mix (Solgent, Republic of Korea) was used for PCR amplification under the following conditions: 95°C for 5 min, 35 cycles of 95°C for 5 min, 50°C–55°C for 5 min, and 72°C for 3 min, and then 72°C for 10 min.

### 2.4 Southern blot analysis

Genomic DNAs of WT and PNG were extracted using a previously reported protocol (Jeong Br et al., 2002). 10 μg of genomic DNAs were digested with *Nco*I, *Pst*I, *Kpn*I, and *EcoR*V, and separated by 0.8% agarose gel electrophoresis. After hydrolysis with 0.2 N HCl, denaturation with 0.5 M NaOH and 1.5 M NaCl, and neutralization with 0.5 M Tris-HCl pH 8.0 and 1.5 M NaCl, DNA-fragments were transferred onto a Hybond-N^+^ nylon membrane (GE healthcare, UK) by capillary transfer methods. A 0.4-kb PCR fragment of *aphVII* gene was used as a probe, amplified by primers aph72_L and aph72_R. The probes were labeled and hybridized using the DIG-High Prime DNA Labeling and Detection Starter Kit II (Roche, Germany) following the manufacturer’s instructions. The probe-bound membrane was washed, and the immunological signals were detected using the ChemiDoc system (Bio-Rad, United States of America).

### 2.5 GAPDH expression of PNG by quantitative real-time PCR (qRT-PCR)

GAPDH expression was determined by quantitative real-time PCR (qRT-PCR) and Western blotting. Total RNAs were extracted using the RNeasy Plant Mini kit (Qiagen, Germany) and the RNase-Free DNase Set (Qiagen, Germany) according to the manufacturer’s instructions. To eliminate any residual DNA in the samples, we employed the DNA-free DNase Kit (Ambion, United States of America). cDNAs were synthesized using Superscript III Reverse Transcriptase and oligo (dT)_20_ primers (both from Invitrogen, United States of America). The primers SHK GAPDH_F and SHK GAPDH_R were used to analyze the expressional level of GAPDH mRNA, whereas the primers SHK ACTIN_F and SHK ACTIN_R were used as loading control to amplify the housekeeping gene *ACTIN*. The 20 μL reaction mixture was composed of 20 ng cDNAs, 0.5 μL of 10 μM forward/reverse primer, and 10 μL SsoAdvancd SYBR Green supermix (Bio-rad, United States of America). PCR conditions consisted of the following steps: 95°C for 2 min, 40 cycles of 95°C for 10 s, 52°C for 10 s, and 72°C for 20 s, then 95°C for 10 min, and followed by a melt curve at 65°C–95°C (in 0.5°C increments). Gene expression was determined by 2^−ΔΔCT^ method, provided by the analysis program CFX Manager (Bio-rad, United States of America).

### 2.6 Quantification of the lipid contents

Lipid analysis was conducted after transesterification (Jeon et al., 2021). Cells were harvested at 5,035 × g for 10 min, washed twice with deionized water, and lyophilized. 10 mg of lyophilized biomass was reacted with 2 mL of chloroform-methanol mixture (2:1, v/v) with vigorous mixing for 10 min 1 mL of internal standard (C17:0) dissolved in chloroform (100 mg heptadecanoic acid/200 mL chloroform) was added for gas chromatography (GC) analysis. The extracted lipids were converted into fatty acid methyl esters (FAMEs) via transesterification by adding 1 mL of methanol and 300 μL of sulfuric acids at 100°C for 20 min. Then, 1 mL of 0.3 N sodium hydroxide was added to remove the residual methanol and sulfuric acid, and the mixture was shaken vigorously. After centrifugation 1,644 × g for 10 min, the organic phase (lower layer) was obtanined and filtered using 0.20 μm RC-membrane syringe filter (Sartorius Stedim Biotech, Germany). FAMEs were analyzed by GC (HP5890, Agilent) with a flame-ionized detector and an HP-INNOWAX capillary column (30 m × 0.32 mm × 0.5 μm, Agilent, United States of America). The identification and quantitation of fatty acids were determined by comparison of retention times and peak areas with FAME standards (FAME Mix C8 - C24, Supelco, United States of America).

### 2.7 Transmission electron microscopy

Cells were centrifuged at 1,644 × g for 5 min and washed twice with 0.1 M phosphate buffer. Then, 0.1 M phosphate buffer containing 2.5% glutaraldehyde was used for fixation. After treatment with Epon 812 embedding media, specimens were observed by bio-transmission electron microscopy (bio-TEM) Tecnai G2 Spirit (FEI Co., Netherlands) at Korea Basic Science Institute.

### 2.8 Observation of lipid bodies by confocal microscopy

For detection of lipid bodies, cells were stained by BODIPY 505/515 (1 μM, 4,4-Difluoro-1,3,5,7-Tetramethyl-4-Bora-3a,4a-Diaza-*s*-Indacene, Invitrogen, United States of America) solubilized in 0.2% Dimethyl sulfoxide (DMSO) and incubated under dark condition for 10 min. LSM510 META NLO confocal microscope (Carl Zeiss, Germany) was used to observe the BODIPY stained cells, with an argon laser at 488 nm, 30 mW for excitation and HeNe 543 nm, and 1 mW for emission. Confocal images were taken with ×50 magnification and then analyzed with LSM image browser software (Carl Zeiss, Germany).

### 2.9 Determination of total carbohydrate and protein contents

To analyze the contents of total carbohydrates and protein contents, 5 mg of lyophilized biomass was dissolved in 1 mL of deionized water and mixed on a vortex mixer. Dissolved biomass was reacted with anthrone solution (2 mg anthrone dissolved in 75% (v/v) sulfuric acid) at 100°C for 15 min. After cooling on ice for 5 min, the absorbance of the supernatant was measured at 620 nm and calibrated by glucose standards at 0–240 mg/L. For the determination of protein contents, the Quick Start Bradford Protein Assay (Bio-rad, United States of America) was used following the manufacturer’s instructions. Briefly, dissolved biomass was reacted with 1 N sodium hydroxide at 100°C for 10 min. Then, 20 μL of the sample was mixed with 1 mL of 1× dye reagent and allowed to react for 5 min. Absorbance readings were taken at 595 nm and calibrated using BSA standards, with concentrations varying from 0 to 1 mg/mL.

### 2.10 RNA sequencing

Total RNA was extracted using the RNeasy Plant Mini kit (Qiagen, Germany), and samples were prepared using the Illumina TruSeq RNA Sample Preparation Kit v2 (catalog #RS-122-2001, Illumina, San Diego, CA) following the manufacturer’s instructions. Briefly, mRNA was purified using poly(A) selection. Subsequently, the RNA was chemically fragmented and converted into single-stranded cDNA using random hexamer priming. After the generation of double-stranded cDNA, an A-base was added to the blunt ends to prepare them for the ligation of sequencing adapters. Following size selection of the ligated products, the cDNA fragments containing adapter sequences were amplified via PCR using adapter-specific primers. The library was quantified using a KAPA library quantification kit (Kapa Biosystems, KK4854) according to the manufacturer’s instructions. Each library was then loaded onto an Illumina HiSeq 2000 platform, and high-throughput sequencing was performed to ensure that each sample achieved the desired average sequencing depth. Sequence data with a base pair quality of Q ≥ 20 were extracted using SolexaQA. This trimming process resulted in reads having a mean length of 87.86 bp across all samples, with a minimum length of 25 bp. The trimmed reads were mapped to the reference transcripts of *C. reinhardtii* (v5.5), which were downloaded from the Phytozome database (http://phytozome.jgi.doe.gov/). This mapping was performed using the RNA-seq mapping algorithm implemented in Bowtie2 software (version 2.1.0), allowing a maximum of two mismatches. The number of mapped clean reads for each gene was counted and then normalized using the DESeq package in R (Anders and Huber, 2010) to mitigate bias due to varying sequencing amounts (Supplementary Figure S1).

Differentially expressed genes (DEGs) were identified using significance analysis by the DESeq R library. Genes with at least a two-fold change (either up- or downregulation) were considered significant. All correlation analyses and hierarchical clustering were performed using the AMAP library in R (Lucas, 2014). For the functional annotation of each DEG list, Gene Ontology (GO) analysis was conducted based on the sequence similarity (e-value cut off ≤ 1e^−10^) of proteins in the Gene Ontology database (Ashburner et al., 2000). All the sequencing data has been deposited into NCBI under bioproject PRJNA1081661.

### 2.11 Full genome sequencing and single nucleotide polymorphism (SNP)/InDel analysis

A schematic workflow for the genome sequencing and SNPs/indels analysis was presented in Supplementary Figure S2. Briefly, DNA library preparation and cluster generation were carried out using the TruSeq DNA PCR-Free Sample Preparation Kit, TruSeq Rapid SBS Kit, and TruSeq Rapid PE Cluster Kit, all from Illumina, United States of America. Sequencing of *the C. reinhardtii* DNA libraries was executed on the Illumina HiSeq 2,500 system. A paired-end sequencing approach was employed on the Illumina HiSeq 2,500 to examine SNPs/indels between the WT and the PNG transformant. Short reads from the WT and PNG were processed using the SolexaQA package, adhering to specific quality and length criteria (Probability value: 0.05, Phred quality score: 20, minimum read length: 25 bp). Subsequently, the sequenced data was aligned with the *C. reinhardtii* reference genome v5.5 (available on Phytozome ver 10.1) using Burrows-Wheeler Aligner (BWA). Raw SNPs/indels were identified and extracted using SAMtools and an in-house script. Filtering of detected SNPs/indels was based on set parameters: Read depth of at least 3, Mapping quality of 30 or higher, and Biallelic SNPs/indels. Regions with unmapped or unknown nucleotides were excluded. For genotype determination, a read depth exceeding 90% indicated homozygosity, while a read depth ranging between 40% and 60% suggested heterozygosity. All the sequencing data has been deposited into NCBI under bioproject PRJNA1081661.

## 3 Results and discussion

### 3.1 The construction and overexpression of pCrN1GA vector in *Chlamydomonas reinhardtii*

In *C. reinhardtii*, four isoforms of GAPDH enzymes are identified (Table 1). Localization prediction tools (ChloroP, TargetP, and PredAlgo) suggest distinct cellular locations for these isoforms. GAP1 and GAP2 are predicted to be mitochondrial GAPDHs, whereas GAP3 is identified as a chloroplast GAPDH. GAP4 is anticipated to be a cytosolic GAPDH. Generally, cytosolic GAPDH primarily participates in glycolysis, converting GAP to 1,3-BPG. In contrast, chloroplast GAPDH plays a dual role in both the Calvin cycle and glycolysis, catalyzing the reversible conversion of 1,3-BPG to GAP (Johnson and Alric, 2013). This suggests that GAP3 is integral to central carbon metabolism, including CO_2_ fixation and lipid synthesis. Therefore, in this study, the focus was on overexpressing the endogenous chloroplast GAP3 to explore its impact on these metabolic pathways.

**TABLE 1:** The genetic information of *GAPDH* in *Chlamydomonas*. (M: Mitochondria, C: Chloroplast, O: Others).

Gene ID	Accession	CDS	a.a	CDS function	ChloroP^a^				TargetP^a^						PredAlgo^a^
					Score	cTP	CS-score	cTP-length	cTP	mTP	SP	Other	Loc	RC	
Cre12.g485150.t1.2	XP_001703199.1	GAP1	371	Mostly cytosolic	0.499	-	9.636	10	0.623	0.644	0.006	0.031	M	5	O
Cre07.g354200.t1.2	XP_001702068.1	GAP2	347	Similar with cyanobacteria	0.438	-	−0.477	20	0.004	0.826	0.2	0.172	M	2	O
Cre01.g010900.t1.2	XP_001689871.1	GAP3	374	Subunit A, chloroplast	0.529	Y	10.568	22	0.79	0.111	0.038	0.122	C	2	C
Cre12.g556600.t1.2	XP_001694180.1	GAP4 (GAPN1)	499	Non-phosphorylating	0.467	-	6.092	65	0.069	0.121	0.086	0.815	-	2	O

^a^ The localization of each CDS was predicted using ChloroP (http://www.cbs.dtu.dk/services/ChloroP/), TargetP (http://www.cbs.dtu.dk/services/TargetP/), and PredAlgo (https://giavap-genomes.ibpc.fr/predalgo/).

The linearized pCrN1GA vector, containing the chloroplast *GAP3* gene under the control of the NIT1 promoter, was introduced into C. *reinhardtii*. The integration of the transgene was verified by genomic DNA PCR. This PCR targeted the hygromycin-resistant *aphVII* gene, the transgenic sequence (NIT1 promoter to *GAPDH* gene), and 18S rDNA (SSupplementary Figure S1A; Supplementary Table S1). The *aphVII* and transgenic genes were present in both the vector and transformants but absent in the WT, confirming successful transformation (Figure 1B; Supplementary Figure S3A). Seven transformant strains, designated as PNG transformants, were identified with integrated vector sequences. Subsequent cultivation of these PNG transformants under nitrogen-repleted or depleted conditions revealed varied growth and FAME content results (Supplementary Figure S3B–D). Most transformants (PNG #1 to #6) showed lower growth rates compared to WT under nitrogen-replete conditions and no significant differences under nitrogen-depleted conditions. The FAME contents in these transformants were also similar to each other. However, PNG #7 showed enhanced growth in both nitrogen-replete and depleted conditions. Notably, its FAME content reached 30% on day 4 under nitrogen starvation, significantly outperforming the WT and other transformants (about 20% of FAME content). Consequently, PNG #7 was selected for an in-depth study to elucidate the mechanisms of lipid accumulation under nitrogen deprivation.

To determine the number of transgene copies integrated into the genome of PNG #7, Southern blot analysis was performed using the *aphVII* gene as a probe (Figure 1D). The genomic DNAs were digested with various restriction enzymes (*Nco*I, *Pst*I, *Kpn*I, and *EcoR*V), and multiple bands were observed exclusively in the PNG #7 strain. This pattern suggested that the three copies of the transgenes were integrated into the PNG#7 genome. Quantitative RT-PCR (qRT-PCR) was also conducted to assess GAPDH mRNA levels using primers SHK GAPDH_F and SHK GAPDH_R, with ACTIN mRNA serving as a normalization control (Supplementary Table S1). Six hours post-cultivation under nitrogen-starved conditions, PNG#7 exhibited higher GAPDH expression than WT (Figure 1C). This finding implied that the chloroplast GAPDH transcription is efficiently regulated by the NIT1 promoter under nitrogen-depleted conditions, leading to elevated expression levels. These results confirmed the successful integration of the pCrN1GA vector into the PNG#7 strain and the effective expression of the chloroplast GAPDH (GAP3) under nitrogen starvation conditions. For simplicity, the PNG#7 strain will henceforth be referred to as ‘PNG.’

### 3.2 Growth and lipid analysis of the PNG strain

**FIGURE 2:**
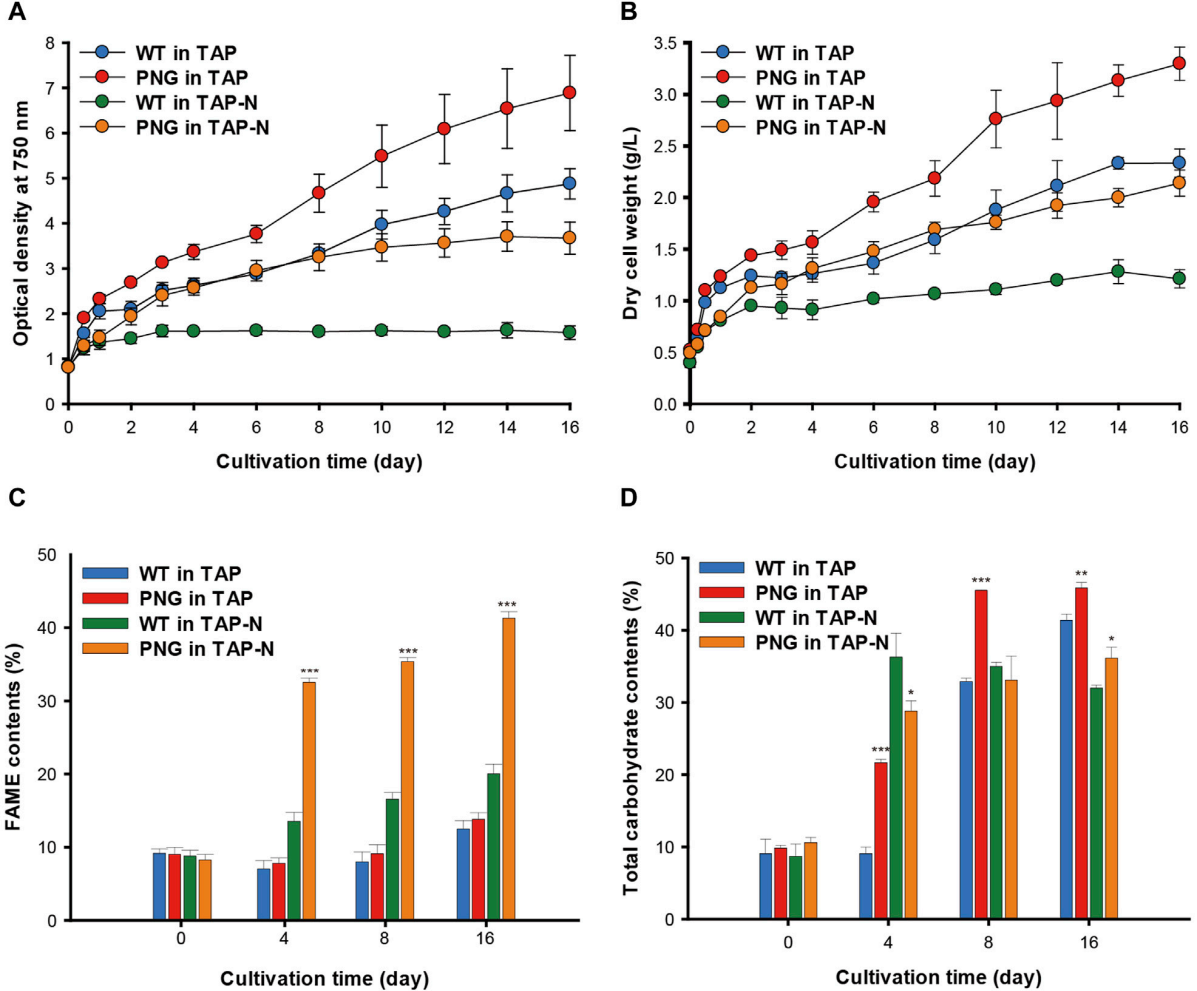
Cultivation of WT and PNG under nitrogen-replete and deplete conditions **(A)** Growth curve based on optical density at 750 nm, and **(B)** Dry cell weight measured for 16 days. **(C)** FAME contents and **(D)** carbohydrate contents of WT and PNG on days 0, 4, 8, and 16. Error bars indicate standard errors obtained from four independent experiments. Significant differences, as determined by the Student’s *t*-test, are indicated by asterisks (*p < 0.05, **p < 0.01, ***p < 0.001).

The phenotype of the PNG strain was investigated under nitrogen-replete and depleted conditions. The PNG and WT strains were cultivated over 16 days, and growth was measured by optical density (OD) and dry cell weight (DCW). Consistent with the initial screening data (Supplementary Figure S3B, C), the PNG strain exhibited higher OD_750 nm_ values than WT under both nitrogen conditions (Figure 2A). This was consistent with the DCW results, with PNG showing a 44% and 76% increase on days 4 and 16 under nitrogen starvation conditions, respectively (Figure 2B).

**FIGURE 3:**
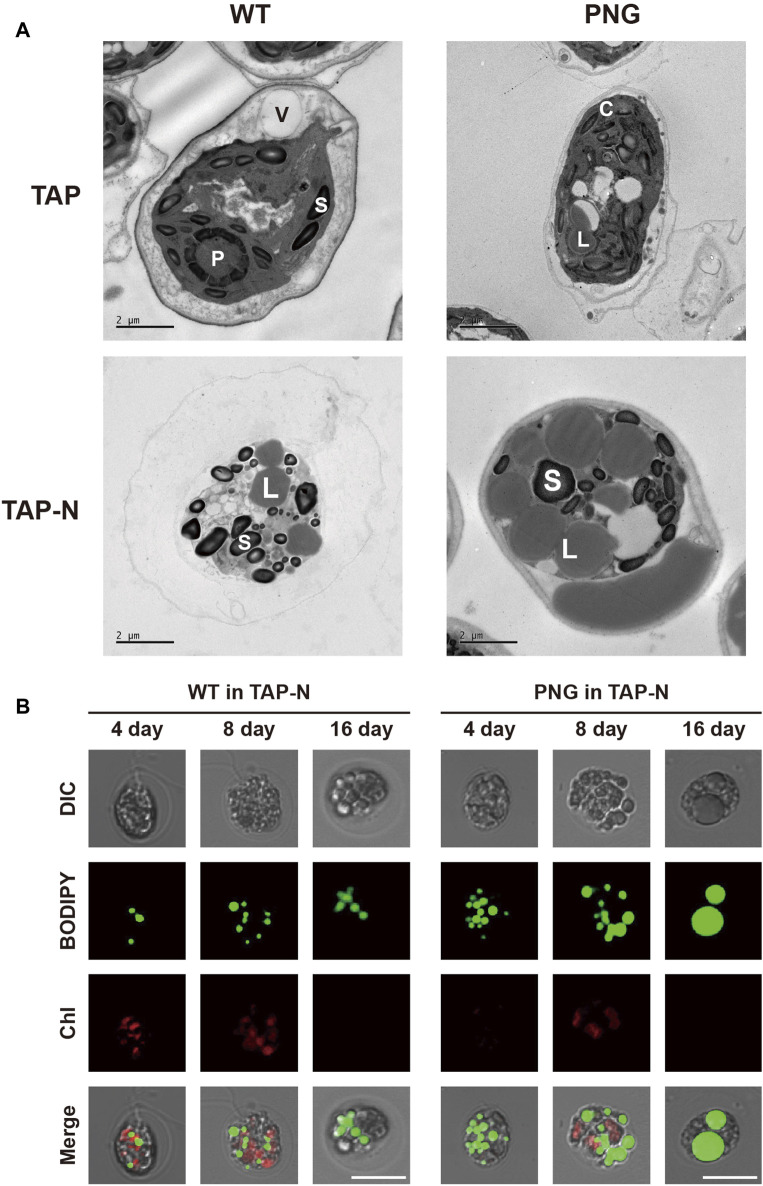
Microscopic observation of lipid droplets in WT and PNG transformants under normal and nitrogen-depleted conditions. **(A)** Transmission electron microscopy (TEM) images taken at 16 days post-induction. V: vacuole, P: pyrenoids, S: starch granule, C: chloroplast, and L: lipid body. **(B)** Confocal microscopy images stained with BODIPY 505/515. The first row shows differential interference contrast (DIC) images. The second row displays BODIPY fluorescence. The third row presents chlorophyll auto-fluorescence, and the fourth row is a merged image of all three. Scale bars represent 10 μm.

Lipid analysis was also conducted over the cultivation period. Under nitrogen-replete conditions, the PNG strain did not exhibit significantly higher lipid production, likely due to low expression of the GAP3 gene by the inducible NIT1 promoter. However, under nitrogen deprivation, the FAME contents in the PNG strain were 32.52% and 41.29% on days 4 and 16, which were 2.4 and 2.1 times higher than WT, respectively (Figure 2C). This result was comparable to the lipid-rich Bafj5 strain previously reported in *C. reinhardtii* (Wang et al., 2009; Yang et al., 2020; Yang et al., 2022). The FAME composition between the two strains was similar (Supplementary Table S2). In terms of FAME yield, considering both DCW and FAME content, the PNG strain outperformed WT by 3.4 and 3.7 times at days 4 and 16 under nitrogen starvation, respectively (Supplementary Figure S4). Interestingly, despite the general inverse relationship between biomass productivity and lipid accumulation (Rodolfi et al., 2009; Williams and Laurens, 2010), PNG exhibited enhanced lipid accumulation and growth, suggesting its potential as an oleaginous strain suitable for biodiesel production. Confocal and transmission electron microscopy further confirmed the high lipid accumulation in PNG, especially under nitrogen starvation. PNG showed an increased number of lipid bodies, which coalesced into larger droplets over time (Figures 3A,B).

Biochemical composition analysis revealed no significant differences in protein content between WT and PNG strains regardless of nitrogen conditions (Supplementary Figure S5). However, the total carbohydrate content varied, with PNG storing more carbohydrates than WT under nitrogen-repleted conditions (Figure 2D). Under nitrogen deprivation, the carbohydrate content in PNG was similar to WT, indicating a potential shift from carbohydrate to lipid, facilitated by the overexpression of GAP3 under the control of the NIT1 promoter.

### 3.3 Transcriptome analysis of PNG transformant

Transcriptome analysis was conducted to elucidate the mechanisms behind lipid accumulation and cellular growth in the PNG transformant. After processing three biological replicates, RNA sequencing data were trimmed, yielding an average of 79.20% usability from the raw data. The pair plot analysis showed that the reproducibility of biological triplicate samples was higher than 0.92, ensuring high reproducibility and reliability of the data (Supplementary Figure S6). These were then annotated to the *C. reinhardtii* genome using the Phytozome database, which identified 8,599 transcripts among a total of 17,741. Remarkably, 92.63% of the sequenced reads mapped to these 17,741 reference transcripts in *C. reinhardtii*.

**FIGURE 4:**
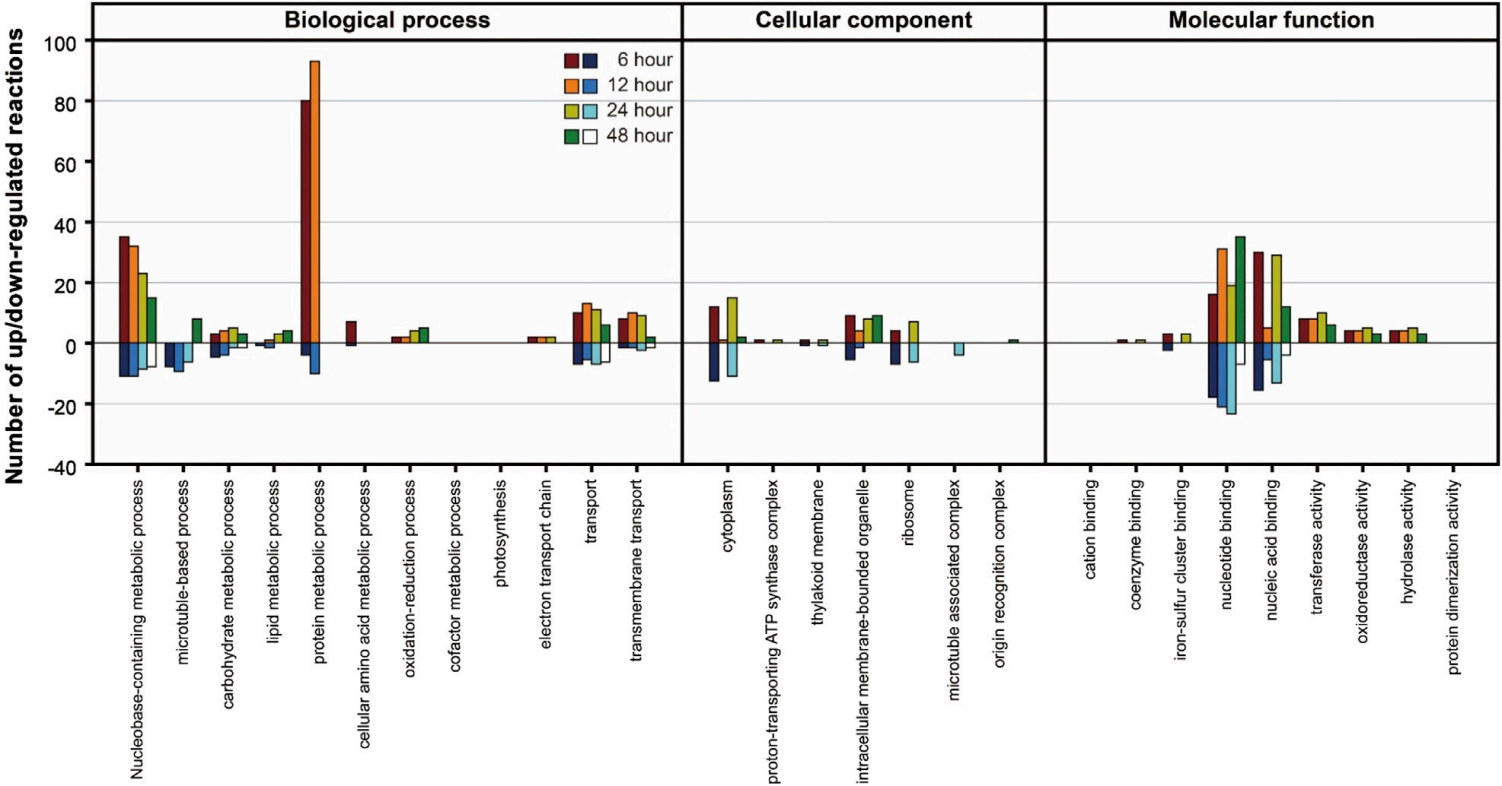
GO analysis of DEGs between PNG and WT under nitrogen-depleted conditions. The *Y*-axis represents the number of reactions that were upregulated or downregulated according to Gene Ontology classifications. Samples were prepared at intervals of 6, 12, 24, and 48 h after nitrogen starvation.

In the Differentially Expressed Gene (DEG) analysis, the gene expression of the PNG strain was primarily examined in comparison to the WT under nitrogen starvation conditions. Significant changes in gene expression over time were identified (Supplementary Table S3), revealing a higher number of upregulated genes in the PNG strain compared to WT. To further understand the biological implications of the DEGs, a Gene Ontology (GO) analysis was conducted (Figure 4). In the biological process category, protein and nucleobase-containing metabolic processes were prominently upregulated in the PNG transformant at 6 and 12 h, but this trend diminished at 24 and 48 h. This pattern suggests an early stage focused on DNA replication and protein expression in the PNG transformant, resulting in better growth relative to WT (Figures 2A,B). In terms of molecular function, a general upregulation of genes related to nucleotide and nucleic acid binding in the PNG strain was observed, suggesting a potential regulation of gene expression through transcription factor binding. This could explain the pronounced phenotypic changes observed.

**FIGURE 5:**
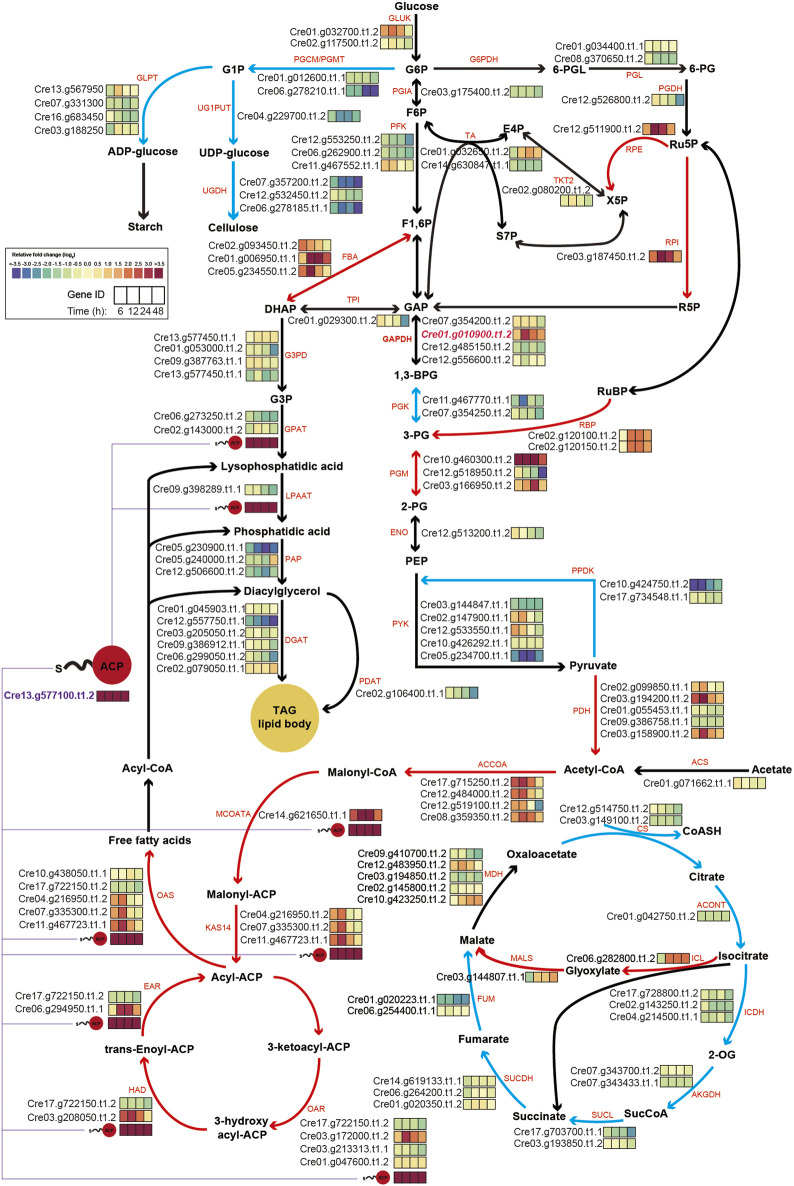
Schematic map of central carbon metabolism, integrating transcriptomics data under nitrogen starvation conditions. The log2-fold changes in the gene expression of central metabolic genes under nitrogen depletion are represented as color-coded boxes in the diagram. A shift towards purple signifies lower expression levels, whereas a shift towards red indicates higher expression levels in PNG transformants relative to WT. Red and blue lines within the figure denote pathways anticipated to be upregulated or downregulated, respectively. ACP, acyl-carrier protein; TAG, triacyl-glycerol; G1P, glucose-1-phosphate; G6P, glucose-6-phosphate; F6P, fructose-6-phosphate; F1,6P, fructose-1,6,-phosphate; GAP, glyceraldehyde-3-phosphate; 1,3-BPG, 1,3- bisphosphoglyceric acid; 3-PG, 3-phosphoglycerate; DHAP, dihydroxyacetone phosphate; G3P, glycerol-3-phosphate; 6-PGL, 6-phosphogluconolactonase; 6-PG, 6-phospho gluconate; Ru5P, Ribulose 5-Phosphate; R5P, Ribose 5-Phosphate; X5P, xylulose-5-phosphate; E4P, Erythrose 4-phosphate; S7P, Sedoheptulose 7-phosphate; 2-PG, 2-Phosphoglyceric acid; PEP, Phosphoenolpyruvic acid; 2-OG, 2-Oxoglutarate; GLPT, glucose-1-phosphate adenylyltransferase; UG1PUT, UTP-glucose-1-phosphate uridylyltransferase; UGDH, UDP-glucose 6-dehydrogenase; PGCM, phosphoglucomutase; GLUK, glucokinase; PGIA, glucose-6-phosphate isomerase; PFK, phosphofructokinase; G6PDH, glucose-6-phosphate dehydrogenase; PGL, 6-phosphogluconolactonase; PGDH, phosphogluconate dehydrogenase; RPE, d-Ribulose-5-phosphate 3-epimerase; TKT2, transketolase 2; TA, transaldolase; RPI, ribose-5-phosphate isomerase; FBA, fructose-bisphosphate aldolase; TPI, triosephosphate isomerase; GAPDH, glyceraldehyde-3-phosphate dehydrogenase; G3PD, glycerol-3-phosphate dehydrogenase; GPAT, glycerol 3 phosphate acyltransferase; LPAAT, lysophosphatidic acid acyltransferase; PAP, phosphatidate phosphatase; DGAT, diglyceride acyltransferase; PGK, phosphoglycerate kinase; PGM, phosphoglycerate mutase; ENO, enolase; RBP, rubisco binding protein; PYK, pyruvate kinase; PPDK, pyruvate, phosphate dikinase, PDH: pyruvate dehydrogenase, PDAT, phospholipid:diacylglycerol acyltransferase; ACS, acetyl-CoA synthase; ACCOA, acetyl-CoA carboxylases; MCOATA, malonyl-CoA-ACP transacylase; KAS14, 3-ketoacyl-ACP synthase; OAR, 3-oxoacyl-ACP reductase; HAD, 3-Hydroxyacyl-CoA dehydrogenase; EAR, enoyl-ACP reductase; OAS, ketoacyl synthases; CS, citrate synthase; ACONT, aconitate hydratase; ICL, isocitrate lyase; MALS, malate synthase, ICDH, isocitrate dehydrogenase; AKGDH, 2-oxoglutarate dehydrogenase; SUCL, succinyl-CoA ligase; SUCDH, succinate dehydrogenase; FUM, fumarate hydratase; MDH, malate dehydrogenase.

To gain a clearer understanding, central carbon metabolism pathways with RNA expression levels based on transcriptome results were mapped (Figure 5). A key observation was the consistently high expression level of the overexpressed chloroplastic GAP3 (Cre01. g010900. t1.2) up to 10-fold throughout the cultivation period under nitrogen starvation conditions, compared to WT. This might be attributed to the integration of the three copies of GAP3 (Figure 1D). This elevated expression of GAP3 appeared to positively influence both the Calvin cycle and glycolysis within the chloroplast, leading to more efficient carbon assimilation. Additionally, the PNG transformant also showed increased gene expression in the glyoxylate cycle (*ICL* and *MALS*), suggesting an augmented malate synthesis and oxaloacetate synthesis. This could potentially enhance gluconeogenesis and improve the overall carbon flux in the organism.

In the lipid synthesis pathway, the gene encoding the acyl-carrier protein (*ACP*, Cre13. g577100. t1.2) was significantly upregulated during the cultivation period. Given the involvement of the ACP in multiple reactions of fatty acid and TAG synthesis from pyruvate, its increased expression likely plays a central role in lipid accumulation in the PNG transformant. Alongside the acyl-carrier protein gene, several other genes related to fatty acid synthesis, including *ACCOA*, *MCOATA*, *KAS14*, *CAR*, *HAD*, *EAR*, and *CAS*, exhibited higher expression levels.

Conversely, genes associated with cellulose syntheses, such as *PGCM/PGMT* (Cre06. g278210. t1.1) and *UGDH* (Cre07. g357200t1.2 and Cre06.278185. t1.1), were significantly downregulated. This indicated that under nitrogen starvation, the PNG strain preferentially channels increased carbon flux, resulting from *GAP3* overexpression, into lipid synthesis pathways while reducing cellulose synthesis, compared to WT.

Likewise, the substantial overexpression of GAP3, resulting from integrating multiple copies, was likely to positively impact carbon assimilation. Although further overexpression of GAP3 could potentially increase lipid production, the complexity of carbon metabolism and the lipid synthesis pathway means that boosting just one gene expression may not significantly improve lipid production. Indeed, in the PNG strain, various transcriptional alterations appear to play a significant role in substantially enhancing lipid production. The underlying mechanisms driving these observed phenotypic and genotypic changes will be detailed in the sections that follow.

### 3.4 SNP/indel analysis: investigating beyond GAPDH overexpression

The intentional overexpression of chloroplast *GAPDH* (*GAP3*) was hypothesized to enhance the Calvin cycle and gluconeogenesis, potentially increasing carbon flux towards lipid synthesis. However, the extensive transcriptomic and phenotypic alterations observed in this study were more profound than expected, suggesting that these changes were not solely due to GAPDH overexpression. Thus, a comprehensive SNP/Indel analysis was conducted to find other genetic factors that might contribute to significant changes in the PNG strain. Interestingly, despite having only three copies of the GAPDH gene integrated (Figure 1D), a surprisingly high number of genetic variations (250 SNPs and 111 Indels) were discovered in the PNG strain (Table 2). This observation may be attributed to the DNA damage and repair mechanisms often triggered by glass-bead transformation, a method known to potentially cause more extensive genetic changes than others, such as electroporation (Pollock et al., 2017). Previous studies have reported significant genetic variations, including large-scale deletions of up to ∼36 kb in *C. reinhardtii*, following this transformation technique (Wang and Spalding, 2006; Dent et al., 2015). The physical stress induced by glass bead transformation, which involves tearing multiple parts of the cell membrane and allowing for the direct contact of exogenous DNA during agitation, could lead to an increased incidence of unexpected cellular DNA alterations (Zhang et al., 2021). However, the specific mechanisms or reasons behind these extensive genetic changes are yet to be fully elucidated, suggesting that the significant number of SNPs and Indels observed in the PNG strain could be a consequence of this method to cause mechanical disruption and subsequent DNA repair processes. Consequently, it is hypothesized that the random mutations induced by the transformation process could contribute to tremendous transcriptomic changes, enhancing lipid production in the PNG strain with the overexpression of *GAP3*.

**TABLE 2:** Comparison of genomic DNA of PNG and WT.

PNG vs. WT	SNP	In/Del
Polymorphic loci^a^	250	111
Ambiguous loci^b^	22,237	1,308
Unknown loci^c^	2,415	723
Filtered loci^d^	4,504	2,056

^a^ Polymorphic loci: Cases where different samples show polymorphism at the same SNP, loci in a comparison.

^b^ Ambiguous loci: Cases where it is difficult to identify polymorphism at the same SNP, loci between comparison samples due to ‘other’ types.

^c^ Unknown loci: Cases where it is difficult to identify polymorphism at the same SNP, loci between comparison samples due to ‘missing data’.

^d^ Filtered loci: Cases where the condition ‘read depth <3′is not met at the same SNP, loci between comparison samples.

## 4 Conclusion

In this study, the PNG transformant was obtained by overexpressing chloroplast *GAPDH* (*GAP3*) in *C. reinhardtii*. This enzyme, which plays a crucial role in the reversible conversion of GAP to 1,3-BPG, is integral to the Calvin cycle for CO_2_ fixation and glycolysis within the chloroplast. As *GAP3* was overexpressed under the control of the nitrogen-inducible NIT1 promoter, the PNG transformant showed a significant increase in lipid production under nitrogen starvation conditions. Transcriptome analysis revealed extensive transcriptional changes in the PNG transformant under these conditions. Notably, the gene encoding the acyl-carrier protein (ACP), involved in various reactions of fatty acid and TAG synthesis, was significantly upregulated. Moreover, enhanced expression of several genes in the pathway from pyruvate to fatty acids synthesis was observed, while genes related to cellulose synthesis were generally downregulated. SNP/Indel analysis further indicated that extensive DNA modifications likely contributed to the profound transcriptomic and phenotypic changes, potentially synergizing with GAPDH overexpression to augment lipid production. While additional studies are needed to fully elucidate the mechanisms underlying the observed increases in biomass and lipid production, the findings indicate that chloroplast *GAPDH* is a key gene for boosting carbon metabolism in microalgae, thereby enhancing lipid production. This study not only advances understanding of microalgal lipid synthesis but also highlights the potential of metabolic engineering strategies in optimizing biofuel precursors.

## Data Availability

The data presented in the study are deposited in the NCBI repository, accession number SRR28131285 ∼ SR28131310 (Bioproject PRJNA1081661). Any further inquiries can be directed to the corresponding author.
