# Supplementary material for: Increasing lipid production in Chlamydomonas reinhardtii through genetic introduction for the overexpression of glyceraldehyde-3-phosphate dehydrogenase
Source: Front Bioeng Biotechnol. 2024 Apr 19;12:1396127. doi: 10.3389/fbioe.2024.1396127 (PMC11066295; doi:10.3389/fbioe.2024.1396127)
Supplement: Supplementary file 1 [file DataSheet1.docx]

Supplementary Material

**Increasing lipid production in *Chlamydomonas reinhardtii* through genetic introduction for the overexpression of *glyceraldehyde-3-phosphate dehydrogenase***

**Sung-Eun Shin^1†^, Hyun Gi Koh^2†^, Kyungmoon Park^2^, See-Hyoung Park^2^, Yong Keun Chang^1^, Nam Kyu Kang^3^***

^1^Department of Chemical and Biomolecular Engineering, Korea Advanced Institute of Science and Technology (KAIST), Daejeon 34141, Republic of Korea

^2^Department of Biological and Chemical Engineering, Hongik University, Sejong 30016, Republic of Korea

^3^Department of Chemical Engineering, College of Engineering, Kyung Hee University, Yongin, 17104, Republic of Korea

*** Correspondence:**Nam Kyu Kang
[nkkang@khu.ac.kr](mailto:nkkang@khu.ac.kr)

**^†^These authors have contributed equally to this work and share the first authorship.**

## Supplementary Figures

**Supplementary Figure S1.**

(a)

(b)

**Supplementary Figure S1.** **Boxplot of gene expression level before and after normalization**. (**a**) Average expression level of the raw data from each sample (**b**) Average expression level of the normalized data from each sample.

**Supplementary Figure S2.**

**
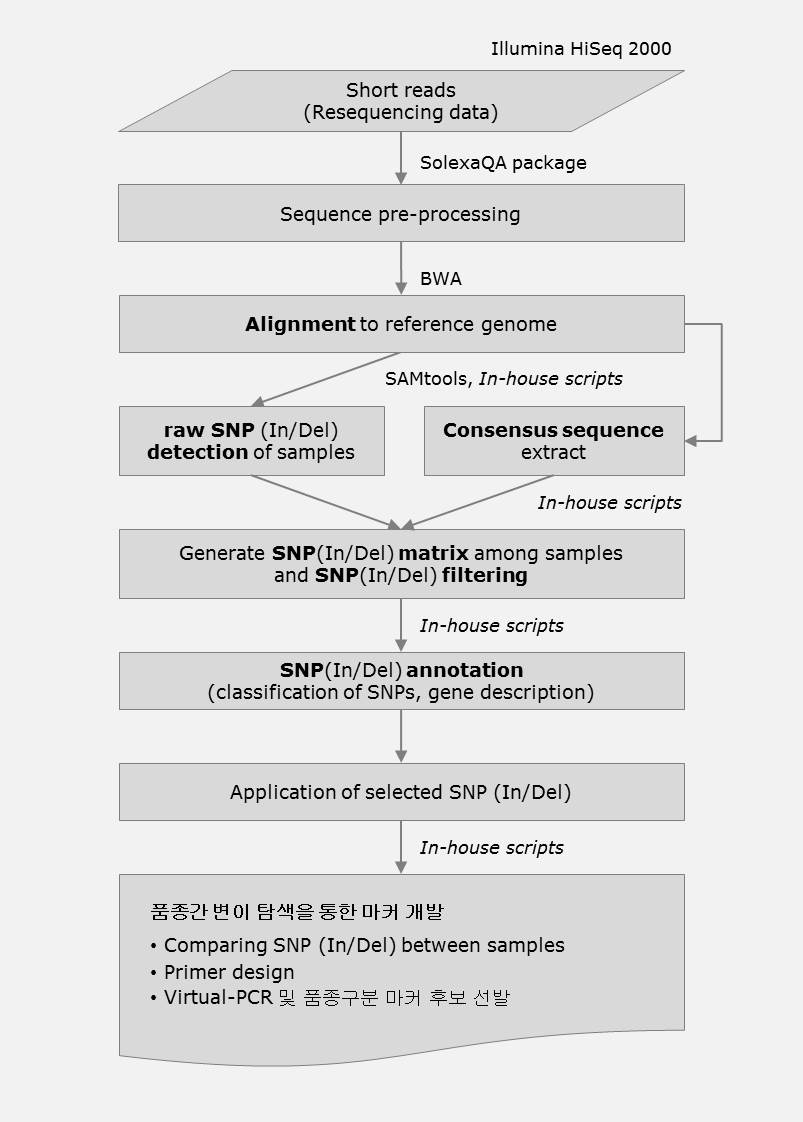
**

**Supplementary Figure S2. A schematic workflow for the genome sequencing and SNPs/indels analysis.**

**Supplementary Figure S3.**


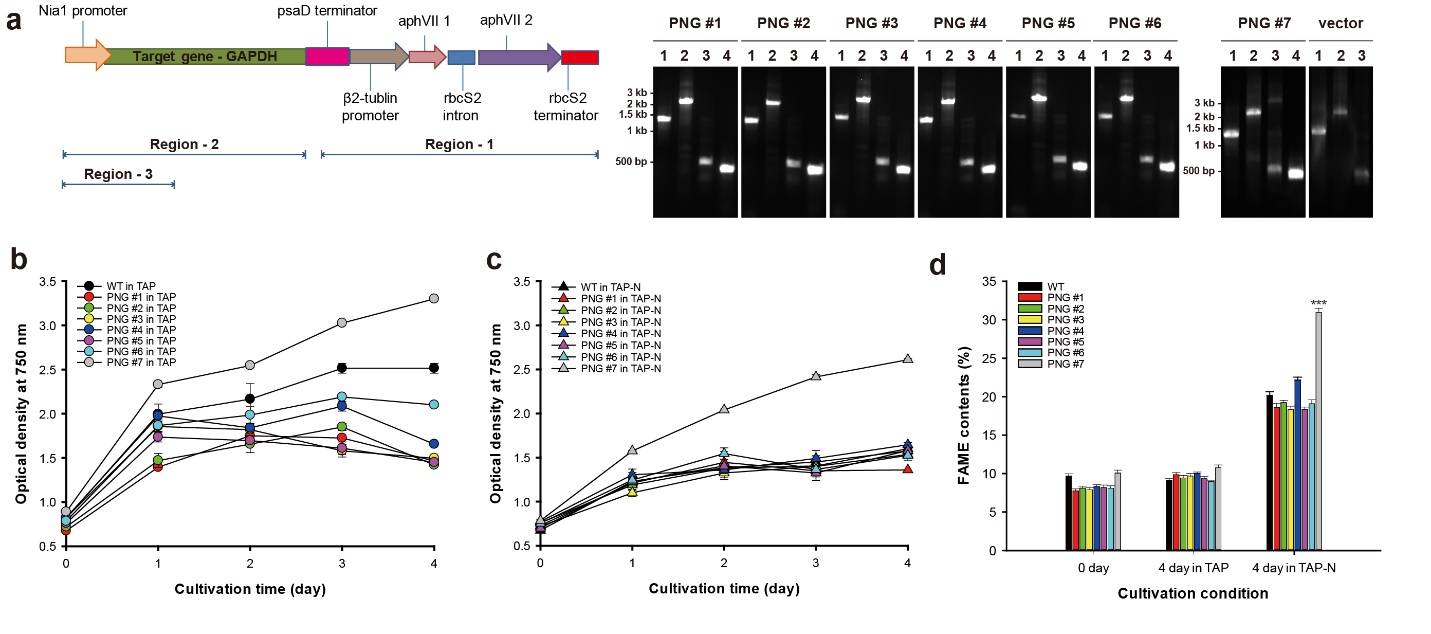


**Supplementary Figure S3. Strain selection and screening cultivation data of pCrN1GA transformants.** (**a**) PCR confirmation of the integrated pCrN1GA vector sequences. Specific primer information for amplification of each region is listed in Supplementary Table 1. (**b**) Growth curve of PNG transformants in TAP medium. (**c**) Growth curve of PNG transformants under TAP medium without ammonium. (**d**) FAME contents of PNG transformants at 4 days of induction. Error bars indicate standard errors obtained from three independent experiments. Significant differences, as determined by Student’s *t*-test, are indicated by asterisks (*P<0.05, **P<0.01, ***P<0.001).

**Supplementary Figure S4.**


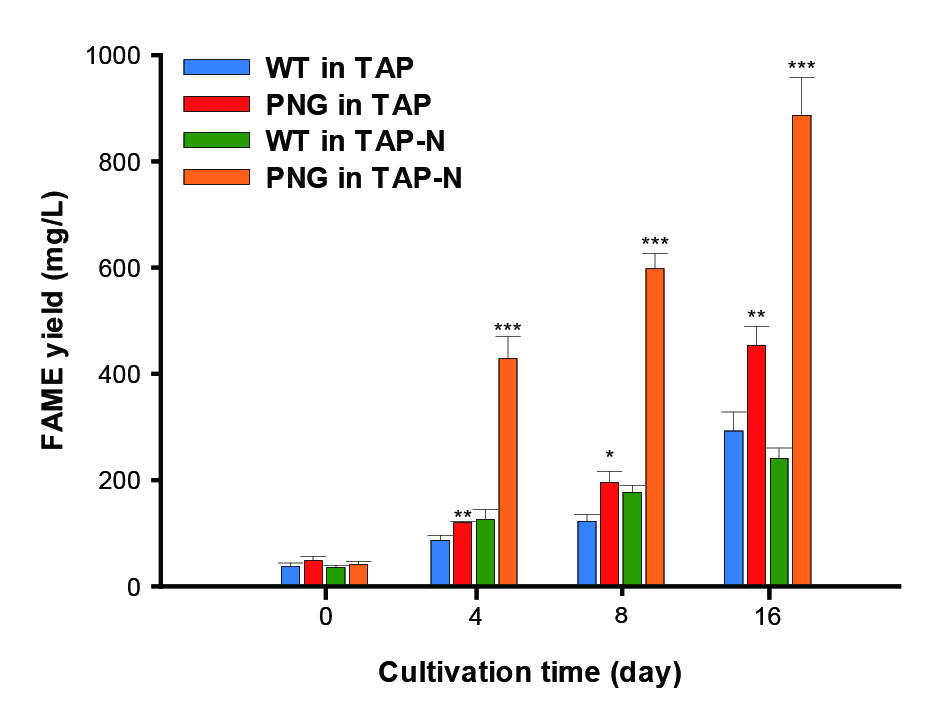


**Supplementary Figure S4. FAME yields of PNG and WT under nitrogen-replete and deplete conditions.** Error bars indicate standard errors obtained from four independent experiments. Significant differences, as determined by the Student’s *t*-test, are indicated by asterisks (**P <* 0.05, ***P* < 0.01, ****P* < 0.001).

**Supplementary Figure S5.**


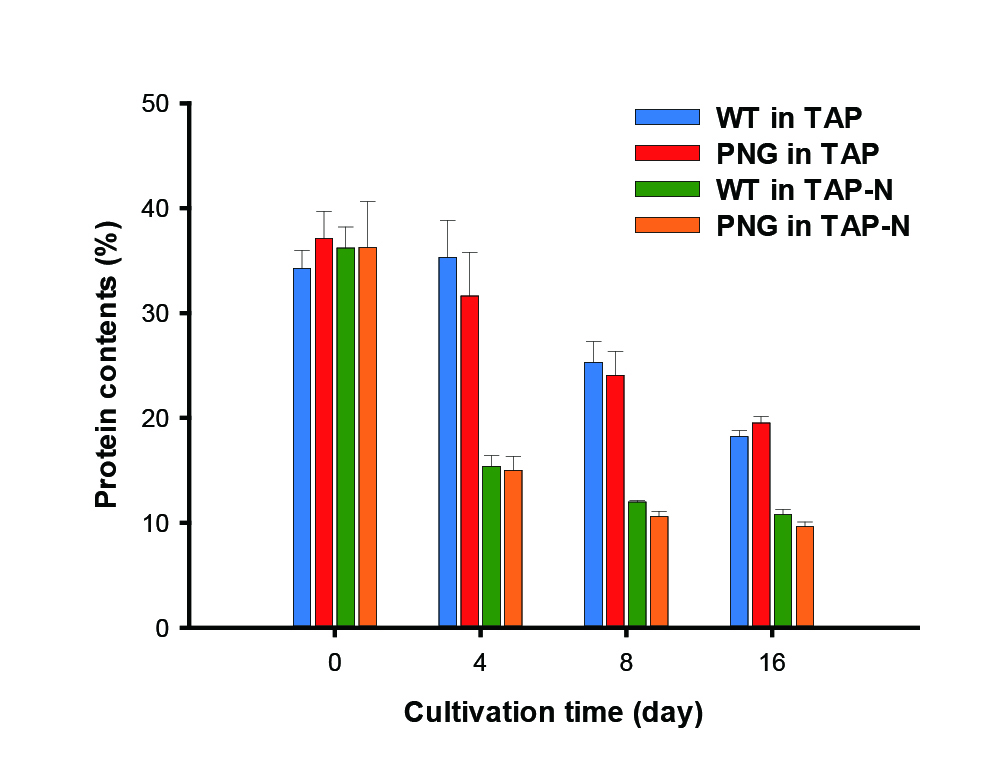
**Supplementary Figure S5. Protein contents of PNG and WT under nitrogen-replete and deplete conditions.** Error bars indicate standard errors obtained from four independent experiments. Significant differences, as determined by Student’s *t*-test, are indicated by asterisks (**P <* 0.05, ***P* < 0.01, ****P* < 0.001).

**Supplementary Figure S6.**


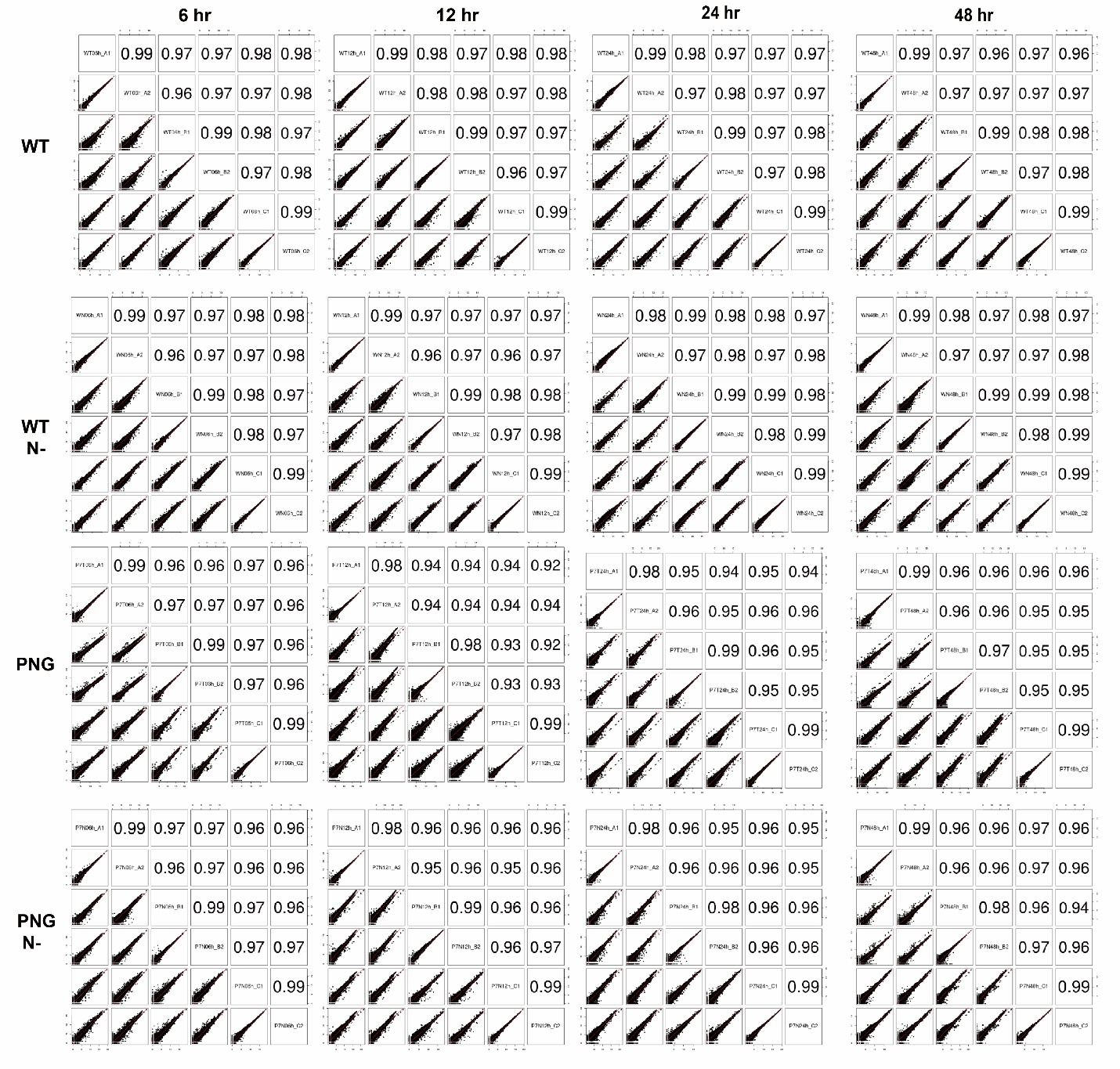


**Supplementary Figure S6.** **Pair Plot Analysis of Data Set Reproducibility.** The average Pearson correlation coefficient is 0.96, with the minimum value exceeding 0.9, indicating high reproducibility.

## Supplementary Tables

**Supplementary Table S1. Primers in this study**

|  | **Primer** | **Sequence 5'-3'** | **Purpose** | **Size (bp)** |
| --- | --- | --- | --- | --- |
|  | NIT_F | GGGGTACCAACCGACCAATCGATAG | Vector construction: *NIT1* promoter | 296 |
|  | NIT_R | CCCAAGCTTCCCGGGACTAGTACTGGCAGGATT |  |  |
|  | GF | GACTAGTATGCAGAAGGTGCGCAG | Vector construction: *GAPDH* gene | 1,977 |
|  | GR | CCCGGGTTATTACGCCACCCACTTC |  |  |
|  | hpt204_L | AGCGAGCTACCAAAGCCATA | PCR confirmation for transformant selection  - hygromycin-resistant gene (region - 1) | 1,375 |
|  | hpt1579_R | TACCGCTTCAGCACTTGAGA |  |  |
|  | NIT_F | GGGGTACCAACCGACCAATCGATAG | PCR confirmation for transformant selection  - transgenic gene (region - 2) | 2,228 |
|  | GR | CCCGGGTTATTACGCCACCCACTTC |  |  |
|  | NIT_F | GGGGTACCAACCGACCAATCGATAG | PCR confirmation for transformant selection  - transgenic gene (region - 3) | 480 |
|  | GMR | GGAGGACACGGCGCTGCCGGT |  |  |
|  | SR6_F | GTCAGAGGTGAAATTCTTGG | PCR confirmation for transformant selection  - 18S rDNA | 380 |
|  | SR9_R | AACTAAGAACGGCATGCAC |  |  |
|  | aph72_L | CCCCCATTCCGAGGTCTTCC | Hygromycin probe for Southern blot | 387 |
|  | aph72_R | AAGGTGAAGGCGAGCAGTTC |  |  |
|  | SHK GAPDH_F | CCAAGGACAAGGACATCC | qRT-PCR for GAPDH mRNA | 145 |
|  | SHK GAPDH_R | ACAATGCCGAACTTCTGC |  |  |
|  | SHK ACTIN_F | CTCTACAACAACATCGTGCT | qRT-PCR for Actin mRNA | 189 |
|  | SHK ACTIN_R | CATTTGCTGGAAGGTGGA |  |  |

**Supplementary Table S2. FAME composition of lipids from WT and PNG strain cultivated under TAP media and nitrogen depleted TAP media**

**(%)**

| **Day** | **Media** | **TAP** | | **TAP-N** | |
| --- | --- | --- | --- | --- | --- |
|  | **Strain** | **WT** | **PNG** | **WT** | **PNG** |
| **Day 4** | myristic acid (C14:0) | 0.23 ± 0.01 | 0.17 ± 0.05 | 0.28 ± 0.08 | 0.12 ± 0.01 |
|  | palmitic acid (C16:0) | 22.88 ± 0.79 | 24.96 ± 0.75 | 39.15 ± 5.28 | 32.44 ± 0.47 |
|  | palmitoleic acid (C16:1) | 0.30 ± 0.01 | 0.23 ± 0.02 | 1.90 ± 0.32 | 0.09 ± 0.00 |
|  | stearic acid (C18:0) | 2.34 ± 0.06 | 2.70 ± 0.07 | n.d. | 3.44 ± 0.06 |
|  | oleic acid (C18:1n9) | 2.21 ± 0.16 | 1.73 ± 0.10 | 21.84 ± 5.61 | 19.86 ± 0.12 |
|  | linoleic acid (C18:2n6) | 3.86 ± 0.30 | 4.36 ± 0.11 | 13.71 ± 2.34 | 13.86 ± 0.25 |
|  | linolenic acid (C18:3n3) | 24.29 ± 0.27 | 24.54 ± 0.36 | 10.09 ± 1.09 | 9.66 ± 0.32 |
|  | etc. | 43.90 ± 0.83 | 41.32 ± 0.60 | 13.04 ± 1.89 | 20.53 ± 0.10 |
| **Day 16** | myristic acid (C14:0) | 1.09 ± 0.66 | 0.76 ± 0.47 | 0.46 ± 0.03 | 0.09 ± 0.03 |
|  | palmitic acid (C16:0) | 36.36 ± 2.37 | 33.69 ± 3.18 | 39.31 ± 6.31 | 36.17 ± 5.04 |
|  | palmitoleic acid (C16:1) | 1.42 ± 0.61 | 1.37 ± 0.58 | 1.76 ± 0.13 | 2.51 ± 0.57 |
|  | stearic acid (C18:0) | n.d. | n.d. | n.d. | 1.18 ± 2.05 |
|  | oleic acid (C18:1n9) | 13.32 ± 3.48 | 12.28 ± 8.12 | 15.49 ± 2.64 | 18.77 ± 12.38 |
|  | linoleic acid (C18:2n6) | 9.03 ± 2.67 | 10.76 ± 3.81 | 10.42 ± 0.63 | 15.00 ± 0.52 |
|  | linolenic acid (C18:3n3) | 10.56 ± 4.98 | 15.20 ± 4.88 | 12.61 ± 1.65 | 7.64 ± 1.43 |
|  | etc. | 28.22 ± 7.48 | 25.94 ± 8.83 | 19.96 ± 3.57 | 18.63 ± 14.45 |

**Supplementary Table S3. DEG statistics of PNG transformant based on transcriptome under nitrogen starvation**

| Cultivation time | Regulation pattern | Number of DEGs | Number of annotated DEGs |
| --- | --- | --- | --- |
| 6 hour | Up | 412 | 217 |
|  | Down | 132 | 93 |
| 12 hour | Up | 443 | 247 |
|  | Down | 210 | 131 |
| 24 hour | Up | 453 | 268 |
|  | Down | 135 | 85 |
| 48 hour | Up | 480 | 288 |
|  | Down | 96 | 54 |
